# Association between Sleep Timing and Weight Status among 14- to 19-Year-Old Adolescents in Wuhan, China

**DOI:** 10.3390/ijerph17165703

**Published:** 2020-08-07

**Authors:** Xinge Zhang, Yanan Chen, Rui Zhang, Justin B. Moore, Haotian Ruan, Jialin Fu, Guiyu Qin, Xinru Yu, Zeyu Hou, Qin Cheng, Xiaoyu Hu, Siqi Zhang, Rui Li

**Affiliations:** 1 School of Health Sciences, Wuhan University, Wuhan 430071, China; bryan@whu.edu.cn (X.Z.); chen_yn1210@163.com (Y.C.); Novamadeus@icloud.com (H.R.); Fjl0708@whu.edu.cn (J.F.); qingyu@whu.edu.cn (G.Q.); yxr1223@whu.edu.cn (X.Y.); fredhou117@163.com (Z.H.); 2017302280009@whu.edu.cn (Q.C.); 2017302280010@whu.edu.cn (X.H.); izsq20190409@163.com (S.Z.); 2 College of Life Sciences, South-Central University for Nationalities, Wuhan 430074, China; zhangrui@mail.scuec.edu.cn; 3 Department of Family & Community Medicine, Wake Forest School of Medicine, Winston-Salem, NC 27157, USA; jusmoore@wakehealth.edu; 4 Department of Epidemiology & Prevention, Wake Forest School of Medicine, Winston-Salem, NC 27157, USA; 5 Department of Implementation Science, Wake Forest School of Medicine, Winston-Salem, NC 27157, USA; 6 Global Health Institute, Wuhan University, Wuhan 430071, China

**Keywords:** sleep timing, obesity, adolescents

## Abstract

This study examined the cross-sectional and longitudinal association of sleep timing with weight status in 14- to 19-year-old adolescents in Wuhan, China. A prospective school-based study was conducted in Wuhan, China between 28 May and 29 September 2019. Data on sociodemographic information, academic performance, diet, mental health status, physical activity, sleep characteristics, body weight, and height were collected. A linear regression model and binary logistic regression model were performed. A total of 1194 adolescents were included in the analysis. Adolescents who woke up before 05:45 had higher body mass index (BMI) Z-score (odds ratio (OR) with 95% confidence interval (CI) = 1.28 (1.05, 1.57), *p* = 0.02) and higher odds of overweight/obesity (odds ratio (OR) with 95% confidence interval (CI) = 1.74 (1.10, 2.76), *p* = 0.02) at baseline after fully adjustment for covariates, compared with those who woke up after 05:45. Longitudinal data showed a nonsignificant association between waking up time and change in BMI Z-score (*p* = 0.18). No association of bedtime with weight status was observed in this sample after full adjustment (*p* > 0.1). Earlier waking up time might contribute to overweight and obesity in adolescents; however, more data are needed to test and elucidate this relationship.

## 1. Introduction

Overweight and obesity are defined as abnormal or excessive fat accumulation caused by the energy imbalance between calories consumed and calories expended [1]. The prevalence of overweight and obesity has been continually increasing worldwide, especially in children and adolescents aged 5–19 [1]. Overweight and obesity are considered as a major cause of many serious diseases including diabetes mellitus, stroke, and cancer [1]. Overweight and obesity in adolescence often extends into adulthood and thus relates to adulthood morbidity and mortality [2]. Beside the health burden, obesity was estimated to account for between 0.7% and 2.8% total health care expenditure of each country worldwide, and obese patients were found to accrue medical costs that were approximately 30% higher than their normal-weight peers [3]. Given the high prevalence, serious health consequences, and huge economic burden associated with overweight and obesity, identifying modifiable risk factors, understanding the etiology and preventing their development are urgent.

Sleep plays a key role in human health. Many animal and epidemiologic human studies have demonstrated the adverse health effects of sleep problems including short sleep duration, poor sleep quality, and various sleep disturbances [4]. As an important component of sleep, the sleep timing, usually measured by bedtime and waking up time, has also been related to some health consequences. Previous studies related sleep timing to hypertension, diabetes, recurrent falls, and osteopenia [4]. Recently, an increasing number of studies suggested that sleep timing might be predictive of overweight and obesity.

Later bedtime and earlier waking up time were shown to be associated with higher body mass index (BMI) and risk of overweight/obesity [5,6,7], and their links with greater severity of overweight were also found in individuals with overweight and obesity [8,9]. However, these associations attenuated or did not reach statistical significance after controlling for confounding factors in other samples or studies [10,11,12,13], and even reversed in some [14,15]. The relationship between sleep timing and weight status has been explored in adults, early adolescents, preschool children, and infants, showing varied results across different age groups [6,14,16], but studies in adolescents aged between 14 to 19 years remain scarce, despite evidence that sleep timing is highly age-dependent [17]. Previous studies predominantly examined the cross-sectional association between sleep timing and weight status, and their results might be weaker because obesity might be also predictive of increased sleep difficulties so that may lead to disorders in sleep timing [18]. We need higher levels of evidence such as analyses based on longitudinal data.

Thus, this study was designed to evaluate the cross-sectional and longitudinal association of sleep timing with baseline weight status and subsequent BMI Z-score change at 4-month follow-up, in a sample of 14- to 19-year-old Chinese adolescents.

## 2. Materials and Methods

### 2.1. Study Population

This is a prospective school-based study conducted in the No.1 Jiangxia High School located in Wuhan, China, beginning on 28 May 2019. Informed consent forms and questionnaires were handed out to all adolescents (*n*= 2395) enrolled in the school, to collect baseline data on sociodemographic variables, academic performance, diet, mental health status, physical activity, sleep characteristics, body weight, and height. Participants were provided no incentive for participation. Follow-up data on body weight and height were collected on 29 September 2019. We received 1984 completed questionnaires, of which 790 were excluded due to—(1) lack of data on bedtime or waking up time (*n* = 127); (2) lack of data on age, sex, body weight, or height (*n* = 103); (3) extreme z-scores (BMI Z-score < −5 or BMI Z score > 5) according to World Health Organization (WHO) growth charts (*n* = 97); (4) lack of data on other variables (*n* = 463). Among the 1194 adolescents included in the cross-sectional analysis, 468 had complete BMI Z-score data at the 4-month follow-up.

The study was conducted in accordance with the Declaration of Helsinki. All procedures were approved by Wuhan University Ethics Board (ethical approval code: 2019YF2056) and the school administrators.

### 2.2. Assessment of Sleep Characteristics

The Chinese version of Pittsburgh Sleep Quality Index (PSQI) was used to measure the sleep characteristics of students [19]. According to this questionnaire, sleep duration was measured using the question, “During the past month, how many hours of actual sleep did you get at night? (this may be different than the number of hours you spend in bed)”, and “≥6.5”. Sleep quality was assessed by “During the past month, how would you rate your sleep quality overall?” The response options were “Very good”, “Fairly good”, “Fairly bad”, and “Very bad”, but only a very limited number of participants reported “very bad” sleep quality, so we divided sleep quality into three groups—“Very good”, “Fairly good”, and “Poor”. We evaluated bedtime and waking up time using the following questions—“During the past month, when have you usually gone to bed at night?” and “During the past month, when have you usually got up in the morning?” Bedtime was divided into three groups—“Before 23:30”, “23:30–24:00”, and “After 24:00”. Waking up time was divided into two groups—“Before 05:45”, and “After 05:45”. Sleep onset latency was assessed by the question—“During the past month, how long (in minutes) has it usually take you to fall asleep each night?”, and it was divided into three groups (minutes)—“<15”, “≥15 to <30”, and “≥30”. Sleep efficiency was calculated as follows:

(Number of hours slept/Number of hours spent in bed) ×100% = (Number of hours slept/ (Waking up time – bedtime)) ×100%(1)

and it was divided into three groups—“>85%”, “75–84%”, and “<75%”.

### 2.3. Covariate Assessment

Sociodemographic indicators—using a structured questionnaire, information on age, gender, grade, residence (Rural or Urban), and household annually income were obtained. The response options of annual household income included “Less than 30,000 RMB”, “30,000–50,000 RMB”, “50,000–100,000 RMB”, “100,000–300,000 RMB”, and “More than 300,000 RMB”.

Academic performance—students were asked to report their rank on the latest examination and the number of their classmates, based on which, academic performance was calculated and divided into two groups—“Top 20%” and “Bottom 80%”.

Diet—we used a food frequency table and the question, “During the past week, how often did you eat the following food?” to assess the food frequency per week. The options were—“0”, “1–3 times/week”, “4–6 times/week”, “1 time/day”, “2 times/day”, “3 times/day”, and “More than or equivalent to 4 times/day”. The food items listed in the table included fruits, vegetables, fried food, meats, popped food, dairy, beverages, eggs, fast foods, nuts, beans, grains, seafood, and deserts.

Mental health—the Chinese version of the Center for Epidemiological Studies-Depression Scale (CES-D) was used to detect the level of depression [20]. A higher score indicated higher level of depression. The Chinese version of Rosenberg Self-Esteem Scale was used to assess self-esteem [21]. Higher score suggested higher self-esteem. The Simplified Chinese version of the 10-item Perceived Stress Scale (SCPSS-10) [22] was applied to measure the perceived stress of participants. A higher score indicated a higher level of perceived stress.

Physical activity—the validated Chinese version of the Physical Activity Questionnaire (PAQ) was used to assess the physical activity level of students [23]. A higher score indicated a higher level of physical activity.

### 2.4. Outcomes

Age, sex, and BMI (weight in kg/height in m^2^) were used to compute BMI z-score using WHO AnthroPlus [1]. Weight status was divided into two categories—Underweight/Normal (BMI z-score < 1) and Overweight/Obese (BMI z-score ≥ 1). BMI Z-score and odds of overweight/obesity were considered as outcomes of cross-sectional analysis, and BMI Z-score change during the four-month follow-up was the outcome of prospective analysis.

### 2.5. Statistical Analysis

Descriptive analyses of the baseline characteristics, including sociodemographic variables, academic performance, diet, physical activity, mental health, and sleep characteristics, by baseline weight status were conducted. The t test, rank sum test, and Pearson’s χ^2^ test were applied to detect the differences in these baseline characteristics between students with BMI Z-score ≥ 1 (Overweight/Obese) and those with BMI Z-score < 1 (Underweight/Normal).

Odds ratios (ORs) and 95% confidence intervals (CIs) for both crude and adjusted by potential confounders were calculated to quantify the magnitude of the cross-sectional relationship between—(a) bedtime, (b) waking up time and—(a) the odds of overweight/obesity, (b) BMI Z-score, using a binary logistic regression model and a linear regression model, respectively. The prospective association between—(a) bedtime, (b) waking up time and the change value of BMI Z-score during the 4-month follow-up was also evaluated using a linear regression model. Model 1 included age and gender. Model 2 included Model 1 plus grade, residence, annual family income, academic rank, intake frequency per week of fruit, fried food, vegetables, meat, puffed food, dairy, beverages, eggs, fast food, nuts, beans, sea food, wheat, and dessert, physical activity score, and mental health status (perceived stress, self-esteem, and depression). Model 3 included model 2 plus sleep duration, sleep quality, sleep efficiency, and sleep onset latency. Variance inflation factor (VIF) was calculated and VIF > 5 was considered as collinearity.

We further compared baseline food frequency across the waking up time group and assessed the relationships between waking up time and other sleep characteristics including sleep duration, sleep efficiency, sleep onset latency, and sleep quality. Analyses were conducted by Stata version 15.0 (StataCorp, College Station, TX, USA).

## 3. Results

### 3.1. Baseline Characteristics

Baseline characteristics of students by weight group are summarized in Table 1. Among 1194 adolescents included in baseline analysis, 207 adolescents with overweight and obesity accounted for 24.87%. They presented significantly different characteristics of gender, residence, the intake frequency of fried food, dairy, beverages, eggs, and physical activity score, compared with underweight/normal students (*p* < 0.05). The level of perceived stress was also slightly different (*p* = 0.09). In this sample, most of students (82.83%) reported a sleep duration less than 6.5 h, and nearly a quarter reported their sleep duration less than 5.5 h. More than a third of students (35.59%) reported poor sleep quality, 3.10% students reported a sleep efficiency lower than 75%, 15.83% students usually spent more than thirty minutes to fall asleep on the bed. Nearly half of the students went to bed between 23:30 and 24:00 in the evening, and 17.34% students woke up before 05:45 in the morning. Except for waking up time (*p* = 0.04), no significant difference in sleep duration, sleep quality, sleep efficiency, sleep onset latency, and bedtime was observed across weight groups at baseline.

### 3.2. Cross-Sectional Association of Sleep Timing with Weight Status

A total of 1194 adolescents were included in the cross-sectional analysis. We first utilized BMI Z-score as the dependent variable to assess its association with the sleep timing measures. The results are presented in Table 2. No association between bedtime and BMI Z-score was found in any of the four models, neither for bedtime group nor per hour later of bedtime. Compared with students who woke up after 05:45, those waking earlier, before 05:45, were more likely to have a higher BMI Z-score (OR with 95% CI in model 3 = 1.28 (1.05, 1.57), *p* = 0.02). Each hour of later waking up time was significantly related to a lower BMI Z-score (OR with 95% CI in model 4 = 0.75 (0.58, 0.99), *p* = 0.04).

We next took the odds of overweight/obesity as the outcome to test its relationship with sleep timing, using a binary logistic regression model (Table 3). Participants with later bedtime had higher odds of overweight/obesity, but no significant association was found. Compared with students who woke after 05:45, those who woke before 05:45 presented higher odds of overweight/obesity (OR with 95% CI in model 4 = 1.74 (1.10, 2.76), *p* = 0.02). Per hour later of waking up time presented a similar association with odds of overweight/obesity as waking up time group (OR with 95% CI in model 4 = 0.56 (0.31, 1.04), *p* = 0.07).

### 3.3. Longitudinal Association of Sleep Timing with Weight Status

We evaluated the association between sleep timing measures and the change in value of BMI Z-score in a 4-month follow-up (Table 4). The BMI Z-score of students with a bedtime after 24:00 increased over 4 months when compared to those who went to bed before 23:30, but the relationship was nonsignificant (OR with 95% CI in model 2 = 1.10 (0.98, 1.23), *p* = 0.09; OR with 95% CI in model 3 = 1.14 (1.00, 1.29), *p* = 0.05), but the association attenuated after adjustment for sleep characteristics in model 4 (OR with 95% CI = 1.13 (0.94, 1.37), *p* = 0.20). We observed no any association of waking up time with the change value of BMI Z-score in this longitudinal analysis.

### 3.4. Association of Waking up Time with Baseline Food Frequency

Because waking up time was found to be associated with weight status at baseline, we compared baseline food frequency across waking up time groups to explore possible mechanisms. As Table 5 shows, adolescents with an earlier waking up time presented a higher intake frequency of beverages and fast food.

### 3.5. The Associations of Baseline Waking up Time and BMI Z-Score with other Sleep Parameters

To explore possible relationships between waking up time and other sleep characteristics, we developed a linear regression model taking waking up time as the dependent variable (Table 6). We can infer that sleep duration (OR with 95% CI = 1.19 (1.16 1.22), *p* < 0.001) and worse sleep quality (OR with 95% CI for “fairly good”= 1.06 (1.02, 1.11), *p* = 0.008; for “not good” = 1.08 (1.03, 1.14), *p* = 0.001) were positively associated with waking up time, and sleep efficiency (OR with 95% CI = 0.31 (0.25, 0.37), *p* < 0.001) and sleep onset latency (OR with 95% CI = 0.98 (0.97, 0.99), *p* = 0.002) were inversely associated with waking up time. No significant associations between sleep parameters and BMI Z-score were found and no significant collinearity was detected (VIF < 5) in Table 2, Table 3 and Table 4, which indicated that waking up time might be a predictor of BMI Z-score, independent of other sleep characteristics.

## 4. Discussion

In this sample, earlier waking up time was found to be significantly associated with a higher cross-sectional BMI Z-score and odds of overweight/obesity, independent of sleep duration and other sleep parameters, while we did not observe a longitudinal association with change in BMI Z-score at 4-month follow-up. Bedtime presented a potential link with weight measures, but those associations were weakened after adjustment for confounding factors, especially sleep characteristics.

Previous epidemiological studies regarding waking up time and weight status were limited and their results varied across different population groups. No significant association was found in healthy adults [24] and toddlers aged 12 to 26 months [25]. A cross-sectional study in adults with intellectual deficiency reported significantly higher odds of obesity in the late-rise group, compared with early-rise group [26]. Zhou et al. found that later accelerometer-measured weekday waking up time predicted higher BMI and waist circumstance among 8- to 12-year-old children with obesity [9]. Beside later waking up time, Scharf et al. suggested that early rising related to a higher odds of obesity among 5-year-old children, Olds et al. found a significant higher adjusted odds ratio for obesity in late-bed/early-rise group than that in late-bed/late-rise group [27], which were consistent with our results. In Chinese adolescents, we observed a negatively linear association between waking up time and BMI Z-score in a cross-sectional analysis, and the same trend in longitudinal analysis based on data of 4-month follow-up.

The mechanism underlying the association between waking up time and weight status is unknown. Some evidence supports that earlier waking up time is related to less physical activity [28], shorter sleep duration [29], poorer sleep outcomes [30], and longer screen time [31], all of which were associated with higher BMI and odds of obesity. In a randomized crossover study conducted by McNeil et al., the earlier waking up time group presented significantly enhanced explicit wanting and liking for high- relative to low-fat foods and increased fasting and post standard breakfast appetite ratings including desire to eat, hunger, and prospective food consumption, compared with the control group and delayed waking up time conditions [32]. Based on the baseline data, we indeed observed a significant association of waking up time with sleep duration, sleep quality, sleep efficiency, and the frequency of beverage and fast food consumption. Interestingly, no association was found between these variables and baseline BMI Z-scores in this sample (Table S2), and the association of waking up time with physical activity was nonsignificant (Table S1), reducing the possibility that the findings were accounted for by other factors related to sleep, physical activity, and diet, which may predict weight status. In addition to behavioral changes and appetite factors, waking up time has also been observed to be related to circadian disruption, which might result in the dysfunction of glucose metabolism, leading to heavier weight status [14], however, no experimental evidence currently exists. Our longitudinal analysis did not find a significant association between waking up time and change in BMI Z-score, which may be due to the short 4-month follow-up, which might be not long enough to detect the longitudinal relationship. It was also possible that there was no causal association between early waking up time and weight status, and their significant cross-sectional relationship might be just an artifact of the influence of other potential factors on obesity. Thus, this link between early waking up time and weight status that we found in Chinese adolescents requires more evidence and further explication.

We did not find a significant association with weight status and bedtime in this sample, which is consistent with some studies [10,25], but not others [33,34,35]. This discrepancy can be partially explained by the various assessments of sleep timing, diverse measurements and definitions of weight status, confounding factors in the model, and different sample size in analysis. Furthermore, of note is the population we focused on, which is quite different from others, not only the age, but also the developmental stage this population were in. Chinese adolescents are under excessive study pressure from the competition with their peers and obedience to parental aspirations so that they are experiencing notably shorter sleep duration and a later bedtime [36,37]. In an international study, Asian teenagers aged between 13 and 17 years old reported a significantly shorter average sleep duration and later bedtime than their peers in New Zealand, European (44 min for sleep duration and 1 h for bedtime), Māori (36 min for sleep duration and 47 min for bedtime), and Pacific (32 min for sleep duration and 17 min for bedtime) backgrounds [38]. In our study, the majority of students (98.91%) reported a bedtime after 22:30, which means that almost all the students of this sample can be divided into the latest bedtime group in other studies [5]. So, this sample itself was in the group of late bedtime, lacking the comparison with the reference group. In addition, more than four in five of students (82.83%) had a sleep duration less than 6.5 h per night, while the American Academy of Sleep Medicine recommended that teenagers 13 to 18 years of age should sleep 8 to 10 h per 24 h on a regular basis to promote optimal health [39], the short sleep duration presented in our sample might weaken the association between bedtime and weight status. For example, a large scale of study in 14,946 preschool children reported that the association between late bedtime and obesity odds was significant after adjustment for sociodemographic factors, but not after further adjustment for sleep duration [12].

This study expands on previous reports in several ways. (1) We assessed the cross-sectional and longitudinal relationship between sleep timing and weight status among Chinese adolescents, whose sleep pattern is quite different from that of western adolescents. (2) Our results found the potential association between earlier waking up time and weight status among Chinese adolescents and highlighted the need for further research. (3) Bedtime might be unrelated to weight status in Chinese adolescents who are preparing for their College Entrance Exam, and relevant policies or interventions for obesity should be carefully applied in this population.

The primary limitation in our study was that we measured sleep characteristics using a self-reported questionnaire rather than objective measurement such as actigraphy, considering it was demonstrated that self-reported sleep parameters overestimated objectively measured sleep, although under-reporting is relatively lower in Chinese population [40], its effect on results could not be ignored. Besides, in spite of the prospective study design, it might be argued that because the period of follow-up was only four months, we failed to assess the long-term association. In addition, the association of sleep timing with weight status might be sensitive to the difference between weekdays and weekends [41], while we did not distinguish the characteristics in weekdays and weekends. Furthermore, we did not explore the mediation of chronotype (refers to sleep timing and diurnal preferences) on the relationship between sleep timing and weight status [42], and further research is needed to work on its role.

## 5. Conclusions

Our findings highlight the association of waking up time with weight status in Chinese adolescents, indicating that earlier waking up time might also contribute to the development of overweight and obesity. However, more evidence is needed to test and elucidate this relationship.

## Figures and Tables

**Table 1:** Baseline characteristics of participants by weight group.

Characteristics	All (*n* = 1194)	Underweight/Normal BMI Z-Score < 1 (*n* = 987)	Overweight/Obese BMI Z-Score ≥ 1 (*n* = 207)	*p*
**Age (years), mean ± SD ^a^**	16.26 ± 0.96	16.27 ± 0.96	16.21 ± 0.97	0.41
**Gender, *n* (%) ^b^**				<0.001
Boys	659 (55.19)	503 (50.96)	156 (75.36)	
Girls	535 (44.81)	484 (49.04)	51 (24.64)	
**Residence, *n* (%) ^b^**				0.02
Urban	698 (58.46)	562 (56.94)	136 (65.70)	
Rural	496 (41.54)	425 (43.06)	71 (34.30)	
**Grade, *n* (%) ^b^**				0.18
Tenth	426 (35.68)	345 (34.95)	81 (39.13)	
Eleventh	508 (42.55)	432 (43.77)	76 (36.71)	
Twelfth	260 (21.78)	210 (21.28)	50 (24.15)	
**Annual family income, *n* (%) ^b^**				
Less than 30,000 RMB	108 (9.05)	82 (8.31)	26 (12.56)	
30,000–50,000 RMB	226 (18.93)	192 (19.45)	34 (16.43)	
50,000–100,000 RMB	421 (35.26)	349 (35.36)	72 (34.78)	
100,000–300,000 RMB	379 (31.74)	317 (32.12)	62 (29.95)	
More than 300,000 RMB	60 (5.03)	47 (4.76)	13 (6.28)	
**Academic performance, *n* (%) ^b^**				
Top 20%	277 (23.20)	232 (23.51)	45 (21.74)	
Bottom 80%	917 (76.80)	755 (76.49)	162 (78.26)	
**Frequency of foods per week, median (min, max)**				
Fruits ^c^	3 (1, 7)	3 (1, 7)	3 (1, 7)	0.58
Fried foods ^c^	2 (1, 7)	2 (1, 7)	2 (1, 7)	0.02
Vegetables ^c^	4 (1, 7)	3 (1, 7)	4 (1, 7)	0.32
Meats ^c^	4 (1, 7)	4 (1, 7)	4 (1, 7)	0.16
Popped food ^c^	2 (1, 7)	2 (1, 7)	2 (1, 7)	0.60
Dairy ^c^	4 (1, 7)	3 (1, 7)	4 (1, 7)	0.02
Beverages ^c^	2 (1, 7)	2 (1, 7)	2 (1, 7)	0.009
Eggs ^c^	3 (1, 7)	3 (1, 7)	3 (1, 7)	0.001
Fast food ^c^	2 (1, 7)	2 (1, 7)	2 (1, 7)	0.61
Nuts ^c^	2 (1, 7)	2 (1, 7)	2 (1, 7)	0.54
Beans ^c^	2 (1, 7)	2 (1, 7)	2 (1, 7)	0.86
Grains ^c^	4 (1, 7)	4 (1, 7)	5 (1, 7)	0.02
Seafood ^c^	2 (1, 7)	2 (1, 7)	2 (1, 7)	0.77
Desserts ^c^	2 (1, 7)	2 (1, 7)	2 (1, 7)	0.19
**Mental health status, median (min, max)**				
Perceived stress ^c^	30 (10, 50)	30 (10, 50)	29 (10, 50)	0.09
Self-esteem ^c^	23 (6, 35)	23 (6, 34)	23 (11, 35)	0.88
Depression ^c^	14 (0, 80)	14 (0, 60)	14 (0, 74)	0.88
Physical activity, median (min, max)	1.95 (0.93, 4.84)	1.94 (0.93, 4.84)	2.14 (0.99, 4.13)	0.01
**Sleep duration (hours), *n* (%) ^b^**				0.48
<5.5	286 (23.95)	230 (23.30)	56 (27.05)	
≥5.5 to <6	194 (16.25)	161 (16.31)	33 (15.94)	
≥6 to < 6.5	509 (42.63)	420 (42.55)	89 (43.00)	
≥6.5	205 (17.17)	176 (17.83)	29 (14.01)	
**Sleep quality, *n* (%) ^b^**				0.71
Very good	211 (17.67)	171 (17.33)	40 (19.32)	
Fairly good	558 (46.73)	466 (47.21)	92 (44.44)	
Not good	425 (35.59)	350 (35.46)	75 (36.23)	
**Sleep efficiency, *n* (%) ^b^**				0.29
>85%	1026 (85.93)	851 (86.22)	175 (84.54)	
75–84%	131 (10.97)	109 (11.04)	22 (10.63)	
<75%	37 (3.10)	27 (2.74)	10 (4.83)	
**Sleep onset latency (min), *n* (%) ^b^**				0.14
<15	768 (64.32)	640 (64.84)	128 (61.84)	
≥15 to <30	237 (19.85)	200 (20.26)	37 (17.87)	
≥30	189 (15.83)	147 (14.89)	42 (20.29)	
**Bedtime, *n* (%) ^b^**				0.98
Before 23:30	462 (38.69)	383 (38.80)	79 (38.16)	
23:30–24:00	552 (46.23)	456 (46.20)	96 (46.38)	
After 24:00	180 (15.08)	148 (14.99)	32 (15.46)	
**Wakeup time, *n* (%) ^b^**				0.04
Before 05:45	207 (17.34)	177 (17.93)	30 (14.49)	
After 05:45	987 (82.66)	810 (82.07)	177 (85.51)	

SD: standard deviation. ^a^ t test; ^b^ Pearson’s χ2 test; ^c^ rank sum test.

**Table 2:** Cross-sectional association between sleep timing and body mass index (BMI) Z-score (*n* = 965).

Timing	Model 1 OR (95% CI)	*p*	Model 2 OR (95% CI)	*p*	Model 3 OR (95% CI)	*p*
**Bedtime**						
Before 23:30	Reference		Reference		Reference	
23:30–24:00	1.07 (0.93, 1.23)	0.38	1.12 (0.96, 1.32)	0.16	1.05 (0.86, 1.28)	0.65
After 24:00	0.95 (0.78, 1.15)	0.58	0.92 (0.74, 1.16)	0.50	0.79 (0.56, 1.12)	0.19
Per hour later	0.99 (0.91, 1.08)	0.85	1.01 (0.91, 1.11)	0.91	1.12 (0.89, 1.40)	0.33
**Waking up time**						
Before 05:45	1.16 (0.98, 1.38)	0.09	1.28 (1.05, 1.55)	0.02	1.28 (1.05, 1.57)	0.02
After 05:45	Reference		Reference		Reference	
Per hour later	0.78 (0.63, 0.97)	0.02	0.76 (0.59, 0.98)	0.03	0.75 (0.58, 0.99)	0.04

CI: confidence interval; OR: odds ratio. Model 1: adjusted for age and sex; Model 2 adjusted for Model 1 plus grade, residence, annual family income, academic performance, intake frequency of fruit, fried food, vegetables, meat, puffed food, dairy, beverages, eggs, fast food, nutss, bean, sea food, wheat, and dessert, physical activity score, perceived stress, self-esteem, and depression; Model 3: adjusted for Model 2 plus sleep duration, sleep quality, sleep efficiency, and sleep onset latency.

**Table 3:** Cross-sectional association of sleep timing with odds of overweight/obesity (*n* = 965).

Timing	Model 1 OR (95% CI)	*p*	Model 2 OR (95% CI)	*p*	Model 3 OR (95% CI)	*p*
**Bedtime**						
Before 23:30	Reference		Reference		Reference	
23:30–24:00	1.06 (0.76, 1.48)	0.73	1.13 (0.76, 1.67)	0.54	1.10 (0.68, 1.78)	0.70
After 24:00	1.21 (0.76, 1.92)	0.43	1.11 (0.64, 1.91)	0.72	1.02 (0.45, 2.35)	0.96
Per hour later	1.08 (0.86, 1.34)	0.51	1.08 (0.83, 1.41)	0.55	1.04 (0.62, 1.74)	0.88
**Waking up time**						
Before 05:45	1.52 (1.03, 2.23)	0.04	1.75 (1.12, 2.72)	0.01	1.74 (1.10, 2.76)	0.02
After 05:45	Reference		Reference		Reference	
Per hour later	0.56 (0.35, 0.91)	0.02	0.56 (0.32, 0.99)	0.046	0.56 (0.31, 1.04)	0.07

CI: confidence interval; OR: odds ratio. Model 1: adjusted for age and sex; Model 2 adjusted for Model 1 plus grade, residence, annual family income, academic performance, intake frequency of fruit, fried food, vegetables, meat, puffed food, dairy, beverages, eggs, fast food, nuts, beans, sea food, wheat, and dessert, physical activity score, perceived stress, self-esteem, and depression; Model 3: adjusted for Model 2 plus sleep duration, sleep quality, sleep efficiency, and sleep onset latency.

**Table 4:** Prospective association between sleep timing and change in BMI Z-score (*n* = 468).

Timing	Model 1 OR (95% CI)	*p*	Model 2 OR (95% CI)	*p*	Model 3 OR (95% CI)	*p*
**Bedtime**						
Before 23:30	Reference		Reference		Reference	
23:30–24:00	0.99 (0.92, 1.07)	0.80	0.99 (0.91, 1.08)	0.78	0.98 (0.88, 1.10)	0.76
After 24:00	1.08 (0.97, 1.21)	0.15	1.14 (1.00, 1.29)	0.05	1.13 (0.94, 1.37)	0.20
Per hour later	1.02 (0.96, 1.08)	0.51	1.05 (0.98, 1.12)	0.14	1.07 (0.92, 1.24)	0.37
**Waking up time**						
Before 05:45	0.93 (0.84, 1.03)	0.18	0.94 (0.83, 1.06)	0.30	0.92 (0.82, 1.05)	0.21
After 06:15	Reference		Reference		Reference	
Per hour later	1.04 (0.92, 1.18)	0.52	1.06 (0.93, 1.22)	0.38	1.11 (0.96, 1.29)	0.18

CI: confidence interval; OR: odds ratio. Model 1: adjusted for age and sex; Model 2 adjusted for Model 1 plus grade, residence, annual family income, academic performance, intake frequency of fruit, fried food, vegetables, meat, puffed food, dairy, beverages, eggs, fast food, nuts, beans, sea food, wheat, and dessert, physical activity score, perceived stress, self-esteem, and depression; Model 3: adjusted for Model 2 plus sleep duration, sleep quality, sleep efficiency, and sleep onset latency.

**Table 5:** Comparisons in food frequency across the waking up time group (*n* = 1194).

Foods	Before 05:45		After 05:45		*p*
	Mean	Median (Min, Max)	Mean	Median (Min, Max)	
Fruits	3.32	3 (2, 4)	3.15	3 (2, 4)	0.11
Fried foods	2.06	2 (1, 2)	2.13	2 (1, 2)	0.33
Vegetables	3.88	4 (3, 5)	3.76	4 (2, 5)	0.29
Meats	3.97	4 (3, 5)	4.06	4 (3, 5)	0.51
Popped foods	2.21	2 (1, 2)	2.24	2 (2, 2)	0.39
Dairy	3.63	3 (2, 4)	3.64	4 (2, 4)	0.78
Beverages	2.26	2 (1, 3)	2.44	2 (2, 3)	0.007
Eggs	3.24	3 (2, 4)	3.22	3 (2, 4)	0.84
Fast food	1.77	2 (1, 2)	1.91	2 (1, 2)	0.002
Nuts	1.89	2 (1, 2)	1.94	2 (1, 2)	0.26
Beans	2.74	2 (2, 3)	2.65	2 (2, 3)	0.93
Grains	2.12	2 (1, 2)	2.10	2 (1, 2)	0.96
Seafood	4.38	4 (3, 6)	4.37	4 (3, 6)	0.84
Desserts	2.35	2 (2, 2)	2.41	2 (2, 3)	0.053

**Table 6:** Baseline relationships between waking (*n* = 1194).

Categories	Waking up Time		BMI Z-Score	
	OR (95% CI)	*p*	OR (95% CI)	*p*
Sleep duration	1.19 (1.16, 1.22)	<0.001	1.01 (0.92, 1.11)	0.89
Sleep efficiency	0.31 (0.25, 0.37)	<0.001	0.99 (0.45, 2.19)	0.98
Sleep onset latency	0.98 (0.97, 0.99)	0.002	1.00 (0.99, 1.01)	0.12
Sleep quality				
Very good	Reference		Reference	
Fairly good	1.06 (1.02, 1.11)	0.008	0.95 (0.79, 1.14)	0.55
Not good	1.08 (1.03, 1.14)	0.001	0.91 (0.74, 1.11)	0.35

CI: confidence interval; OR: odds ratio.

## Supplementary Materials

The following are available online at https://www.mdpi.com/1660-4601/17/16/5703/s1, Table S1: Linear regression model for risk factors of wake time, Table S2: Linear regression model for risk factors of BMI Z-score.
