# Supplementary material for: Association between Sleep Timing and Weight Status among 14- to 19-Year-Old Adolescents in Wuhan, China
Source: Int J Environ Res Public Health. 2020 Aug 7;17(16):5703. doi: 10.3390/ijerph17165703 (PMC7460288; doi:10.3390/ijerph17165703)
Supplement: Supplementary file 1 [file ijerph-17-05703-s001.pdf]

Table S1. Linear regression model for risk factors of wake time.

Explanatory Variables	OR with 95% CI	*p*
Age (years), mean ± SD	1.00 (0.97, 1.03)	0.930
Gender		
Boys	Reference	
Girls	0.96 (0.92, 0.99)	0.022
Residence		
Urban	Reference	
Rural	1.08 (1.04, 1.11)	<0.001
Grade		
Grade ten		
Grade eleven	1.04 (0.99, 1.09)	0.157
Grade twelve	1.05 (0.96, 1.14)	0.282
Annual family income		
Less than 30,000 RMB	Reference	
30,000-50,000 RMB	1.04 (0.97, 1.12)	0.264
50,000-100,000 RMB	1.05 (0.99, 1.12)	0.104
100,000-300,000 RMB	1.02 (0.95, 1.08)	0.616
More than 300,000 RMB	1.04 (0.95, 1.15)	0.388
Academic performance		
Top 20%	Reference	
Last 80%	0.96 (0.92, 1.00)	0.034
Frequency of foods per week, median (min, max)		
Fruit	0.98 (0.97, 1.00)	0.016
Fried food	1.00 (0.98, 1.02)	0.678
Vegetable	0.99 (0.98, 1.01)	0.191
Meat	1.01 (0.99, 1.02)	0.331
Puffed food	1.01 (0.99, 1.03)	0.322
Dairy	1.00 (0.98, 1.01)	0.687
Beverage	1.01 (0.99, 1.02)	0.435
Egg	1.00 (0.98, 1.02)	0.998
Fast food	1.01 (0.98, 1.03)	0.472
Nut	1.00 (0.98, 1.02)	0.898
Bean	1.00 (0.98, 1.01)	0.848
Wheat	1.00 (0.99, 1.01)	0.827
Sea food	1.00 (0.99, 1.02)	0.661
Desert	1.00 (0.98, 1.01)	0.691
Mental health status, median (min, max)		
Perceived stress	1.00 (1.00, 1.00)	0.595
Self-esteem	1.00 (1.00, 1.01)	0.825
Depression	1.00 (1.00, 1.00)	0.368
Physical activity, median (min, max)	1.01 (0.98, 1.04)	0.517
BMI Z-score	0.98 (0.97, 1.00)	0.041

BMI: body mass index; CI: confidence interval; OR: odds ratio; SD: standard deviation.

Table S2. Linear regression model for risk factors of BMI Z-score.

Explanatory Variables	OR with 95% CI	*p*
Age (years), mean ± SD	0.85 (0.75, 0.98)	0.021
Gender		
Boys	Reference	
Girls	0.84 (0.71, 0.99)	0.042
Residence		
Urban	Reference	
Rural	0.89 (0.77, 1.04)	0.150
Grade		
Grade ten	Reference	
Grade eleven	1.16 (0.93, 1.44)	0.189
Grade twelve	1.52 (1.07, 2.16)	0.021
Annual family income		
Less than 30,000 RMB	Reference	
30,000-50,000 RMB	0.76 (0.57, 1.02)	0.072
50,000-100,000 RMB	0.81 (0.62, 1.07)	0.133
100,000-300,000 RMB	0.72 (0.55, 0.94)	0.017
More than 300,000 RMB	0.82 (0.55, 1.23)	0.336
Academic performance		
Top 20%	Reference	
Last 80%	1.00 (0.84, 1.19)	0.970
Frequency of foods per week, median (min, max)		
Fruit	0.96 (0.90, 1.02)	0.226
Fried food	1.07 (0.98, 1.16)	0.112
Vegetable	1.02 (0.96, 1.09)	0.458
Meat	0.95 (0.89, 1.01)	0.121
Puffed food	0.98 (0.91, 1.06)	0.640
Dairy	1.04 (0.97, 1.10)	0.277
Beverage	1.00 (0.94, 1.07)	0.952
Egg	1.10 (1.03, 1.17)	0.007
Fast food	1.06 (0.96, 1.17)	0.253
Nut	0.99 (0.92, 1.07)	0.856
Bean	0.93 (0.87, 0.99)	0.028
Wheat	1.05 (1.00, 1.11)	0.072
Sea food	0.97 (0.90, 1.05)	0.481
Desert	0.96 (0.90, 1.03)	0.270
Mental health status, median (min, max)		
Perceived stress	1.00 (0.98, 1.01)	0.772
Self-esteem	1.00 (0.98, 1.02)	0.731
Depression	1.00 (0.99, 1.00)	0.452
Physical activity, median (min, max)	1.17 (1.05, 1.31)	0.006
Wake time	0.75 (0.58, 0.99)	0.041

BMI: body mass index; CI: confidence interval; OR: odds ratio; SD: standard deviation.
